# Detection of Transmission Clusters of HIV-1 Subtype C over a 21-Year Period in Cape Town, South Africa

**DOI:** 10.1371/journal.pone.0109296

**Published:** 2014-10-30

**Authors:** Eduan Wilkinson, Susan Engelbrecht, Tulio de Oliveira

**Affiliations:** 1 Division of Medical Virology, Department of Pathology, Faculty of Medicine and Health Sciences, Stellenbosch University, Tygerberg, Cape Town, Western Cape Province, South Africa; 2 Africa Centre for Health and Population Studies, University of KwaZulu-Natal, Somkhele, KwaZulu-Natal, South Africa; 3 National Health Laboratory Services, Tygerberg Hospital, Cape Town, Western Cape Province, South Africa; 4 School of Laboratory Medicine and Medical Sciences, University of KwaZulu-Natal, Durban, KwaZulu-Natal, South Africa; 5 Research Department of Infection, University College of London, London, the United Kingdom; University of Athens, Medical School, Greece

## Abstract

**Introduction:**

Despite recent breakthroughs in the fight against the HIV/AIDS epidemic within South Africa, the transmission of the virus continues at alarmingly high rates. It is possible, with the use of phylogenetic methods, to uncover transmission events of HIV amongst local communities in order to identify factors that may contribute to the sustained transmission of the virus. The aim of this study was to uncover transmission events of HIV amongst the infected population of Cape Town.

**Methods and Results:**

We analysed *gag* p24 and RT-*pol* sequences which were generated from samples spanning over 21-years with advanced phylogenetic techniques. We identified two transmission clusters over a 21-year period amongst randomly sampled patients from Cape Town and the surrounding areas. We also estimated the origin of each of the identified transmission clusters with the oldest cluster dating back, on average, 30 years and the youngest dating back roughly 20 years.

**Discussion and Conclusion:**

These transmission clusters represent the first identified transmission events among the heterosexual population in Cape Town. By increasing the number of randomly sampled specimens within a dataset over time, it is possible to start to uncover transmission events of HIV amongst local communities in generalized epidemics. This information can be used to produce targeted interventions to decrease transmission of HIV in Africa.

## Introduction

Despite the massive national antiretroviral role out campaign, increased efforts by local and national government agencies, as well as the involvement of various non-governmental agencies in HIV awareness and prevention campaigns, the transmission of HIV still continues at alarmingly high rates within South Africa [1]. Although the total number of new infections in the country decreased from around 650 000 in 1998 to around 320 000 annual new infections in 2009, it still corresponds to more than 850 new infections every day within the country [2].

The transmission of HIV can be divided into two main categories: vertical transmission from mother to child and horizontal HIV transmission through sexual contact or direct exposure to infected blood or blood products. In recent years, great strides have been made in the prevention of vertical transmission of HIV from mother to child. In June 2010, 40 000 HIV positive infants were born to HIV positive mothers, down from 105 000 in 2004 [3], [4].

Since the successes in the national prevention of mother to child transmission (PMTCT) campaign, focus and attention have shifted towards addressing factors, which may be implicated in the sustained transmission of HIV amongst young adults in local communities. The nature and complexity of HIV transmission are difficult to establish and are, therefore, often misunderstood, especially within sub-Saharan African countries, where a multitude of factors may play a role in the transmission of the virus.

Due to recent advances in the field of advanced phylogenetics and evolutionary biology within the last 15 years, we now have a good understanding of the macro-factors, which have shaped the global HIV pandemic. These include findings from studies, which have elucidated the origin of HIV in humans [5]–[7] as well as the introduction and spread of subtype B HIV-1 in America and the rest of the industrialized world [8]. Currently, there is a growing need to gain a better understanding of the HIV epidemic at a micro level in order to identify factors which may cause sustained HIV transmission amongst local communities. In recent years, epidemiologists, scientists and medical professionals have made great strides in establishing the mode and pathway of transmission of several human pathogens, including HIV [9]–[11]. In the case of HIV, there are several key factors which may influence the spread/transmission of the virus, such as, human migration [12], [13], behavioural practices (compounded by religious, social, sexual or cultural factors) [14], [15], viral load [16], male circumcision [16], war & conflict [17], mobility [12] and the existence of other underlying health problems, (such as the other sexually transmitted infections) [18], [19].

Within the Cape Metropolitan area, there are several key factors which may influence the horizontal transmission of HIV, such as high frequency of unsafe sexual contact with multiple sexual partners, large numbers of concurrent partnerships, high frequency of substance abuse (including alcohol and illegal narcotics) within the local communities, high prevalence of other sexually transmitted infections and a large population of men who have sex with men (MSM) [20].

Currently, the best method of identifying and establishing transmission events of HIV between individuals or within a community is through the use of high-resolution phylogenetic methods of HIV sequence data [21]–[23]. Due to the fact that all HIV sequence data from South Africa were derived from a small number of randomly sampled patients over the course of the epidemic, these methods have not to date revealed/identified transmission clusters of HIV.

The aim of the following study was to determine if a longitudinally sampled dataset derived from HIV-1 infected individualsover a 21-year period (1989–2010) was able to shed light on transmission processes involved at the start of the epidemic in Cape Town, South Africa. We hypothesized that even a small sample number at the beginning of the epidemic would allow the identification of local transmission clusters as human migration was severely restricted during the apartheid years. The identification of transmission clusters and their characterization can provide valuable insights into factors which contributed to the origin of HIV transmission in South Africa.

## Methods

### 1. Ethical considerations

This study (Reference number N09/08/221) was approved by the Health Research Ethics Committee (HREC) of Stellenbosch University (IRB0005239). All Tygerberg Virology (TV) study participants provided written informed consent for the collection of samples and subsequent analyses. The older R cohort, which is described in more detail in the next paragraph, samples dating from 1989 to 1992 were obtained with a waiver of informed consent. This HREC complies with the South Africa National Health Act No 612003 and the United States code of Federal Regulations title 45 Part 46. The committee also abides by the ethical norms and principles for research as established by the Declaration of Helsinki, the South African Medical Research Council Guidelines as well as the Department of Health Guidelines.

### 2. Study population and sequence sampling

Within the present study, we collected sequence data (*gag* p24 and reverse transcriptase *pol* (RT-*pol*) previously characterized and sequenced within the Division of Medical Virology (Stellenbosch University), which is associated with the Tygerberg Academic Hospital in the northern suburbs of Cape Town, South Africa. Over the past 20-years, a number of *gag* p24 and RT-*pol* sequences have been characterized from patients attending Tygerberg Hospital. From 1984 to 1994, patient samples submitted for HIV-1 testing were stored as “R” samples (R, Retrovirus). During the period 1998 to 2004, we also collected samples from different clinics in the Cape Town Metropole. These TV samples were obtained from state and private clinics, an informal settlement, sex worker cohorts and the blood transfusion services [24]. The other samples were obtained from a study investigating HIV-associated neurocognitive disorders (HAND) in Cape Town [25]. We have used the abbreviation “JO” to describe these samples, as it corresponds to the initials of the primary investigator of the study from which these genotypes were generated [25]. These sequences were selected for the purpose of the present study. We also collected all possible available information (race, age, sex, sexual orientation, suspected method of infection/transmission, occupation, local community of residence, treatment status and other medical information) about each of the patients from whom these sequences were generated. Due to the possible effect antiretroviral treatment may have on phylogenetic analysis of RT-*pol* sequence data, we only included sequence data from antiretroviral (ARV) treatment naïve patients in the present investigation. We were able to collect 195 *gag* p24 sequences (1989–2010) and 166 RT-*pol* sequences (1989–2009).

### 3. HIV-1 Subtyping and drug resistance mutation screening

Each of the sequences was submitted for HIV subtyping to 2 online subtyping methods, the jpHMM accessible from the Los Alamos National Laboratory web page (http://www.jphmm.gobics.de/) and REGA v 2.0 [26], to insure that only HIV-1 subtype C sequences were included in the phylogenetic analysis. We also submitted all of the RT-*pol* sequences to the southern African mirror of Stanford HIV-drug resistance database [27] to identify any potential major drug resistance mutations, which may have influenced the phylogenetic analysis.

### 4. Multiple sequence alignments and tree inference

A number of subtype C reference *gag* p24 (n = 435) and RT-*pol* (n = 1310) sequences were obtained from the Los Alamos National Laboratory's (LANL) HIV sequence database (http://www.hiv.lanl.gov/content/index). The 435 *gag* p24 reference sequences that were retrieved from the LANL database included all available HIV-1 subtype C sequences for this genetic region. The *gag* p24 reference strains contained 58 sequences that were sampled from other areas of South Africa, 182 from other southern African countries, 194 sequences sampled from other areas of the world including the HXB2 reference stain, which was included for the purpose of rooting the phylogeny. Therefore, the final *gag* p24 dataset contained a total of 628 sequences (193 sequences from the Cape Town dataset and 435 from the reference dataset).

The 1311-*pol* reference strains that were retrieved from the LANL database represent a subset (roughly a third) of all the available homologs in the database. A smaller number of reference strains were used in the figures in order to allow interpretation of a phylogenetic tree in a publication format. Of the 1311 reference strains, 827 were sampled from other areas of South Africa, 291 from other southern African countries, 192 from other areas of the world, including the HXB2 reference strain of HIV-1, which was included for the purpose of rooting the phylogeny. Therefore, the final *pol* dataset contained 1477 sequences (166 sequences from the Cape Town dataset and 1311 reference sequences). Even though the *pol* dataset contained only a third of the number of homologous sequences in Los Alamos HIV Database, we are confident that this sampling strategy still provides enough background signal to infer accurate phylogenies and to identify transmission clusters. We have reconstructed Minimum Evolution (ME) trees with all available homologous sequences from Los Alamos (n>4000 taxa), which provided similar results (data not shown).

Multiple alignments of the *gag* p24 and RT-*pol* sequences, along with reference sequences, were constructed in ClustalW (http://www.clustal.org/clustal2/) [28]. In order to increase the speed of the alignment, a quick tree was employed to guide the alignments. Alignment was exported into Se-Al (http://www.tree.bio.ed.ac.uk/software/seal/) and was manually aligned. Gaps were excluded from the alignment if the gaps were not present in more than 20% of the taxa in each of the alignments. Sequences were manually aligned until a perfect codon alignment was achieved.

### 5. Identifying and testing potential clusters

Phylogenies were inferred under a Bayesian framework implemented in MrBayes v 3.2 [29], following 100 million steps in the Markov Chain with posterior samples being collected every 10 000 iterations in the chain. Convergence in chains was assessed in Tracer v 1.5 (http://www.tree.bio.ed.ac.uk/software/tracer/). Consensus tree topologies were constructed from all the posterior trees that were sampled in the chain in TreeAnnotator v 1.8.0 (http://www.beast.bio.ed.ac.uk). All phylogenies were inferred under the general time reversible (GTR) model of nucleotide substitution [30] and an estimated gamma shape parameter.

Phylogenies were manually examined in FigTree v 1.3.1 (http://www.tree.bio.ed.ac.uk/software/figtree/) to identify potential clusters of Capetonian sequences. Phylogenies were analysed visually in FigTree v 1.3.1 and with the aid of the PhyloType software application interface [31]. Clusters were identified based on the geographical annotation of taxa with all sequences annotated according to their country of origin. All South African sequences were annotated as South African (ZA) with the exception of the sequences in the Cape Town datasets, which were annotated as Capetonian (CPT).

For the PhyloType analysis, the query parameters were set as follows: {Size/Difference: 1} {Separation/Diversity: 0.1} {Size: 5}. Therefore, these parameters would identify any cluster from the same geographical origin greater than or equal to 5 in size and these clusters could not be broken by more than 1 other sequence from other geographical regions. Furthermore, a separation/diversity ration is used to identify clusters with low genetic diversity and that are highly divergent from the rest of the tree topology (long internal branch lengths). Statistical significance was accessed in PhyloType using 1000 shuffling iterations to compute p-values. A p-value cut off ≤0.005 was used in all PhyloType analyses to infer statistical significance for each cluster.

### 6. Time-scaled phylogenies of clusters

Time resolved phylogenies were constructed for each of the identified clusters for which there was statistical support in the PhyloType analysis. Dated tree topologies, evolutionary rates and population growth rates were co-estimated with the implementation of a Bayesian Markov Chain Monte Carlo (MCMC) approach which was executed in BEAST v 1.8.0 (http://www.beast.bio.ed.ac.uk) with a GTR + Gamma model. Two parametric demographic models, a constant population size [32] and exponential growth [33], and one non-parametric Bayesian skyline plot (BSP) [34] were compared under both a strict and relaxed molecular clock assumption. Each of the run parameters was analysed under both fixed and estimated mutation rates. For the RT-*pol* analysis, the mutation rate was fixed at 2.55×10^−3^ mutations/site/year [35] and for the *gag* p24 analysis, the mutation rate was fixed at 3.00×10^−3^ mutations/site/year [36]. Chains were conducted for at least 100×10^6^ generations, and sampled every 10 000 steps. Convergence in each of the runs was assessed on the basis of the effective sample size (EES) after a 10% burn-in, with the use of the software application Tracer v 1.5 (http://www.tree.bio.ed.ac.uk/software/tracer/). Model parameters were compared for each of the clusters by computing Bayes factors (BF) using marginal likelihood and this was implemented in Tracer v 1.5. Uncertainty in the estimation was indicated by 95% highest posterior density (95% HPD) intervals.

We also analysed all the sequences from the two Cape Town datasets under a non-parametric tree model using both relaxed and strict molecular clock assumptions and an estimated mutation rate. Posterior trees were summarized in a target tree with the use of the TreeAnnotator programme (included in the BEAST software package) by choosing the tree with the maximum product of posterior probabilities (maximum clade credibility) after a 10% burn-in was discarded. Trees were examined in FigTree v 1.3.1 and manually edited for better interpretation and visualization.

## Results

### 1. Patient sampling

Sequence data were obtained form antiretroviral naïve patients from the Cape Town region over a 21-year period. These sequences were derived from blood or blood plasma from both male and female patients between the ages of 21 and 61 years of age. These patients represent a large demographic section of the South African/Capetonian population, including Caucasian, African and people from Mixed Race. The majority of the patients became infected through heterosexual contact, two patients became infected through the MSM route and one patient became infected while in prison.

### 2. HIV subtyping and drug resistance testing

All of the 195-*gag* p24 and 166-*pol* sequences in the Cape Town cohorts were submitted to two online subtyping tools. Three of the *gag* p24 isolates were identified as either subtype B or unique intra subtype recombinant forms. The subtyping of the 166-*pol* sequence dataset showed no signs of either non-subtype C isolates or recombinant forms of HIV-1. The testing of the *pol* sequences in our dataset for HIV drug resistance testing identified a small number of drug resistance mutations. Due to the effect that these mutations might have on the reconstruction of evolutionary histories, all codon sites associated with drug resistance mutations were manually deleted from the sequence alignments.

### 3. Sequence alignments

Sequences alignments of these datasets were constructed using ClustalW and manually edited using Se-Al (http://tree.bio.ed.ac.uk/software/seal/). For the Pol dataset we excluded codon positions associated with HIV drug resistance mutations. The end result was two perfect codon alignments. The *gag* p24 dataset was 444 nucleotides (148 amino acids) long and the RT-*pol* dataset contained 1002 nucleotides (334 amino acids).

### 4. Tree construction and cluster identification

Close examination of each of the two Bayesian phylogenies (*gag* p24 and *pol*), revealed two potential clusters of Capetonian sequences in each of the two trees.

**Figure 1:**
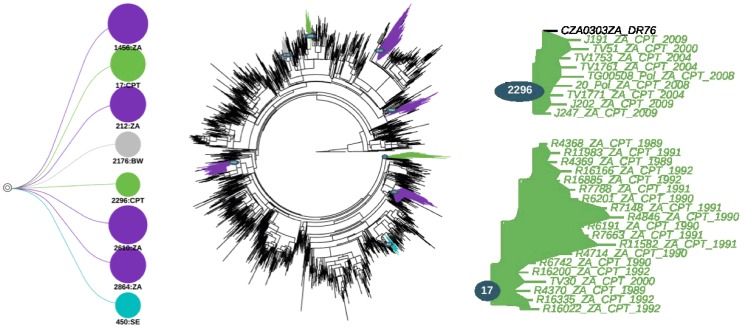
PhyloType schematic of the various clusters found in the analyses of the Bayesian *gag* p24 tree topology. The Bayesian tree topology contains 628 sequences and was constructed in MrBayes with the use of the GTR + Gamma model of nucleotide substitution. The two Capetonian clusters that were identified are presented on the right hand side of the tree while all of the clusters that were identified through the analyses are presented on the left hand side.

Analysis in PhyloType of the two tree topologies, based on strict parameters, identified several clusters for which there was statistical support (p-value ≤0.005). The results of the PhyloType analysis for all identified clusters, including Capetonian clusters, are summarized in Table 1. The analysis of the *gag* p24 tree topology in PhyloType identified 13 clusters in total, two of which were Capetonian clusters (Figure 1). Henceforth, these two Capetonian clusters will be referred to as cluster 20 and cluster 783 (corresponding to their PhyloType ID's). The first cluster (cluster 20) contained a total 18 Capetonian sequences as well as one other sequence from South Africa while the second cluster (cluster 783) contained 11 Capetonian isolates. The posterior support for these two *gag* p24 clusters was 0.673 (cluster 20) and 0.714 (cluster 783) respectively, while the support from the PhyloType shuffling was all statistically significant (p-value ≤0.005).

**Table 1:** The various clusters that were identified in the PhyloType analyses of the *gag* p24 and *pol* Bayesian tree topologies.

	PhyloType ID	Annotation	Size	Difference	Local separation (Sl)	Global separation (Sg)	Diversity (Dv)	Sl/Dv	Sg/Dv	p-values
***gag*** ** clusters**	1263	BR	10	1	0,035	0,177	0,238	0,147	0,744	0,001
	985	BW	8	0	0,058	0,093	0,251	0,231	0,371	0,001
	181	BW	7	0	0,054	0,188	0,177	0,305	1,062	0,001
	475	BW	9	0	0,012	0,142	0,077	0,156	1,844	0,001
	**20**	**CPT**	**19**	**1**	**0,035**	**0,229**	**0,304**	**0,115**	**0,753**	**0,005**
	**783**	**CPT**	**11**	**0**	**0,064**	**0,146**	**0,168**	**0,381**	**0,869**	**0,003**
	1169	IL	20	0	0,105	0,255	0,272	0,386	0,938	0,001
	313	IL	7	0	0,147	0,319	0,153	0,961	2,085	0,001
	893	IL	7	0	0,06	0,132	0,093	0,645	1,419	0,001
	906	IL	16	0	0,023	0,189	0,184	0,125	1,027	0,001
	701	IN	18	1	0,048	0,070	0,240	0,200	0,292	0,001
	1142	KE	13	0	0,044	0,260	0,189	0,233	1,376	0,001
	104	KE	16	0	0,102	0,354	0,129	0,791	2,744	0,001
***pol*** ** cluster**	2176	BW	11	1	0,036	0,252	0,158	0,228	1,595	0,001
	**2296**	**CPT**	**10**	**1**	**0,159**	**0,271**	**0,228**	**0,697**	**1,189**	**0,001**
	**17**	**CPT**	**19**	**0**	**0,052**	**0,062**	**0,437**	**0,119**	**0,142**	**0,001**
	450	SE	10	0	0,163	0,231	0,081	2,012	2,852	0,001
	2610	ZA	24	1	0,17	0,278	0,398	0,427	0,698	0,001
	212	ZA	21	1	0,055	0,177	0,359	0,153	0,493	0,001
	2864	ZA	21	1	0,18	0,233	0,308	0,584	0,756	0,001
	1456	ZA	27	1	0,05	0,169	0,300	0,167	0,563	0,001

ZA – South Africa, SE – Senegal, BW – Botswana, BR – Brazil, CPT – Cape Town, IL – Israel, IN – India, KE – Kenya.

**Figure 2:**
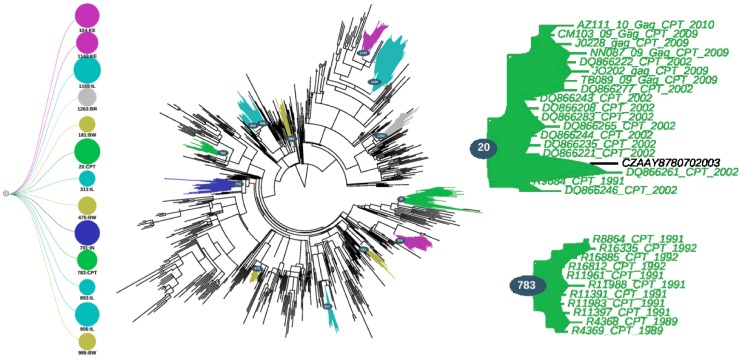
PhyloType schematic of the various clusters found in the analyses of the Bayesian *pol* tree topology. The Bayesian tree topology contains 1477 sequences and was constructed in MrBayes with the use of the GTR + Gamma model of nucleotide substitution. The two Capetonian clusters that were identified are presented on the right hand side of the tree while all of the clusters that were identified through the analyses are presented on the left hand side.

The analysis of the *pol* tree topology in PhyloType identified 8 clusters in total, two of which contained isolates from our Cape Town sequence cohort (Figure 2). These two clusters will henceforth be referred to as cluster 2296 and cluster 17 (corresponding to their PhyloType ID's). The first cluster (cluster 2296) contained a total 9 Capetonian sequences as well as one other sequence from South Africa while the second cluster (cluster 17) contained 19 isolates from Cape Town. The posterior support for these two *pol* clusters was 0.891 (cluster 2296) and 0.815 (cluster 17) respectively, while the support from the PhyloType analyses was all statistically significant (p-value ≤0.005).

Seventeen out of the 51 patients within the cluster were of Mixed Race (33,33%), one was Caucasian (1,96%) and the remaining 33 were of African origin (64,75%). There was an almost equal distribution of male (47,06%) and female (52,94%) patients within each of the clusters. The average age of the patients was 33 years, 10 months and 16 days. The median age of the men was 37 years, 11 months and 12 days and for the women precisely 30 years of age. The large difference in age between the male and female patients within the transmission clusters is consistent with the national variation in age difference in HIV positive young adults within the country (Table 2).

**Table 2:** Available patient information about each of the patients within the identified clusters.

Patient	Sample Date	Sex	Race	Age	Mode of transmission	Available Sequences	Clustering pattern	GenBank Accession Number
R4 368	17-Oct-89	Male	Mixed Race	ND	Hetero	Both	Clusters 17 & 783	KF780993 & KF781090
R4 369	17-Oct-89	Male	Mixed Race	ND	Hetero	Both	Clusters 17 & 783	KF780994 & KF781091
R4 370	17-Oct-89	Male	Mixed Race	ND	Hetero	Both	Cluster 17	KF780995
R4 846	27-Feb-90	Male	Mixed Race	48	Hetero	RT-*pol*	Cluster 17	KF780998
R4 714	01-Feb-90	Male	African	48	Hetero	RT-*pol*	Cluster 17	KF780996
R6 191	23-Oct-90	Female	African	25	Hetero	RT-*pol*	Cluster 17	KF810000
R6 201	25-Oct-90	Male	African	32	Hetero	RT-*pol*	Cluster 17	KF810001
R6 742	14-Dec-90	Female	Mixed Race	55	Hetero	RT-*pol*	Cluster 17	KF810002
R7 148	10-Jul-91	Female	African	25	Hetero	RT-*pol*	Cluster 17	KF781004
R7 663	20-Feb-91	Male	African	23	Hetero	RT-*pol*	Cluster 17	KF781005
R7 788	27-Feb-91	Male	African	61	Hetero	RT-*pol*	Cluster 17	KF781006
R8 864	10-Sep-92	Female	Mixed Race	21	Hetero	*gag* p24	Cluster 783	KF781094
R9 684	12-Jul-91	Male	Mixed Race	23	MSM	*gag* p24	Cluster 20	KF781095
R11 983	18-Nov-91	Male	Mixed Race	32	Hetero	RT-*pol*	Cluster 17	KF780969
R11 391	07-Oct-91	Female	African	37	Hetero	Both	Cluster 783	KF781071
R11 397	27-Feb-90	Male	Mixed Race	48	ND	*gag* p24	Cluster 783	KF781072
R11 582	01-Nov-91	Male	Black	42	Hetero	RT-*pol*	Cluster 17	KF780967
R11 961	15-Nov-91	Male	African	35	Hetero	Both	Cluster 783	KF781074
R11 983	18-Nov-91	Male	Mixed Race	32	Hetero	*gag* p24	Cluster 783	KF781075
R11 988	23-Oct-90	Female	African	25	Hetero	*gag* p24	Cluster 783	KF781076
R16 022	10-Sep-92	Female	Mixed Race	21	Hetero	RT-*pol*	Cluster 17	KF780978
R16 166	21-Sep-92	Male	African	39	Hetero	RT-*pol*	Cluster 17	KF780980
R16 200	23-Sep-92	Male	African	25	Hetero	RT-*pol*	Cluster 17	KF780981
R16 335	02-Oct-92	Male	African	46	Hetero	Both	Clusters 17 & 783	KF780983 & KF781082
R16 812	27-Feb-91	Male	African	61	Hetero	*gag* p24	Cluster 783	KF781085
R16 885	15-Nov-92	Female	Mixed Race	32	Hetero	Both	Clusters 17 & 783	KF780989 & KF781086
DQ866246	08-Apr-02	Male	Caucasian	42	MSM	*gag* p24	Cluster 20	DQ866246
DQ866283	08-May-02	Female	African	29	Hetero	*gag* p24	Cluster 20	DQ866283
DQ866244	09-Apr-02	Male	African	26	Hetero	*gag* p24	Cluster 20	DQ866244
DQ866265	23-Apr-02	Female	African	24	Hetero	*gag* p24	Cluster 20	DQ866265
DQ866208	13-Mar-02	Male	Mixed Race	34	Hetero	*gag* p24	Cluster 20	DQ866208
DQ866261	17-Apr-02	Male	African	27	Hetero	*gag* p24	Cluster 20	DQ866261
DQ866277	06-May-02	Female	African	27	Hetero	*gag* p24	Cluster 20	DQ866277
DQ866243	08-Apr-02	Female	African	26	Hetero	*gag* p24	Cluster 20	DQ866243
DQ866222	19-Mar-02	Female	African	29	Hetero	*gag* p24	Cluster 20	DQ866222
DQ866221	19-Mar-02	Female	Mixed Race	29	Hetero	*gag* p24	Cluster 20	DQ866221
TV30	03-Mar-00	Female	Mixed Race	46	Hetero	RT-*pol*	Cluster 17	KF781048
TV51	21-Aug-00	Male	African	32	Hetero	RT-*pol*	Cluster 2296	KF781053
TV1771	08-Mar-04	Female	African	31	Hetero	RT-*pol*	Cluster 2296	KF781023
TV1761	25-Feb-04	Male	Mixed Race	41	Prison	RT-*pol*	Cluster 2296	KF781017
TV1753	23-Feb-04	Female	African	39	Hetero	RT-*pol*	Cluster 2296	KF781013
JO 191	09-Feb-09	Female	African	ND	Hetero	Both	Cluster 2296 & 20	KF806948 & KF806898
JO 202	09-Mar-09	Female	African	ND	Hetero	Both	Cluster 2296 & 20	KF806954 & KF806905
JO 229	10-Apr-09	Female	African	ND	Hetero	*gag* p24	Cluster 20	KF806920
JO 247	01-Jun-09	Female	Mixed Race	ND	Hetero	RT-*pol*	Cluster 2296	KF806980
AZ1110	22-Jan-10	Female	African	26	Hetero	*gag* p24	Cluster 20	KF793055
NN8709	18-Aug-09	Female	African	27	Hetero	*gag* p24	Cluster 20	KF793085
NM1170	03-Feb-10	Female	African	28	Hetero	*gag* p24	Cluster 20	KF793086
TG0508	08-May-08	Female	African	23	Hetero	RT-*pol*	Cluster 2296	KF793173
NM9009	09-Sep-09	Female	African	31	Hetero	*gag* p24	Cluster 20	KF793083
20 POL	01-Jun-08	Female	African	ND	Hetero	RT-*pol*	Cluster 2296	KF793186

Hetero – Heterosexual; SA – South African National; MSM – Men who have sex with men; ND – No data available; Both – indicating that both *gag* p24 and RT-*pol* sequences were available for this patient.

### 5. Estimating the date of origin of each cluster

The estimated time of the origin of each of the clusters was calculated in BEAST using a Bayesian MCMC approach for both the *gag* p24 and RT-*pol* clusters. The estimated tMRCA's of each of the internal nodes of the identified clusters, as was calculated in the Bayesian inference under the various demographic models of inference, are summarized in Table 3. Bayes factor (BF) calculations for each dataset were conducted in order to identify the “best fitting” model and parameters for each dataset.

**Table 3:** Estimated dates of origin for each of the identified clusters.

Models and Parameters	Cluster 2296			Cluster 17		
	Mean	95% HPD interval	ESS	Mean	95% HPD interval	ESS
Relax.bsp.est	1996,3	1993, 483–1998,740	8482,6	1982,9	1979, 605–1985,724	6207,0
Relax.bsp.fix	1996,8	1994, 292–1999,025	7986,5	1983,4	1980, 784–1985,661	6023,3
Relax.const.est	1995,2	1990, 742–1998,586	7104,5	1978,7	1969, 796–1985,009	4394,0
Relax.const.fix	1995,7	1991, 465–1998,865	7976,2	1979,2	1970, 605–1984,790	5377,1
Relax.expo.est	1995,3	1991, 457–1998,410	8476,5	1980,7	1976, 518–1984,429	3157,5
Relax.expo.fix	1995,9	1992, 134–1998,820	7529,7	1981,6	1977, 804–1984,776	3506,0
Strict.bsp.est	1996,7	1994, 831–1998,555	8458,3	1983,5	1981, 644–1985,223	7899,9
Strict.bsp.fix	1997,7	1996, 385–1999,037	9001,0	1984,5	1983, 271–1985,606	9001,0
Strict.const.est	1996,3	1994, 295–1998,394	7504,9	1982,7	1980, 635–1984,876	7973,2
Strict.const.fix	1997,3	1995, 721–1998,736	9001,0	1983,7	1982, 310–1985,122	8608,9
Strict.expo.est	1996,2	1994, 009–1998,125	8413,1	1982,5	1980, 797–1984,252	7013,4
Strict.expo.fix	1997,3	1995, 811–1998,737	9001,0	1983,8	1982, 441–1985,001	6792,9

Models and Parameters	Cluster 20			Cluster 783		
	Mean	95% HPD interval	ESS	Mean	95% HPD interval	ESS
Relax.bsp.est	1989	1983, 634–1992,500	4310,9	1982,1	1975, 836–1986,344	5708,8
Relax.bsp.fix	1989,5	1984, 327–1992,500	5406,4	1982,2	1976, 337–1986,414	6688,8
Relax.const.est	1983,5	1970, 552–1992,408	6648,3	1978,1	1965, 881–1986,113	7329,5
Relax.const.fix	1984,0	1970, 794–1992,102	6541,4	1978,3	1966, 173–1986,028	7691,9
Relax.expo.est	1985,8	1978, 557–1991,402	6108,8	1981,8	1976, 086–1986,057	6862,0
Relax.expo.fix	1986,4	1979, 204–1991,459	5458,5	1982,0	1976, 662–1986,056	6991,5
Strict.bsp.est	1989,8	1987, 534–1991,499	9001,0	1984,2	1981, 758–1986,368	8303,7
Strict.bsp.fix	1990,4	1988, 713–1991,500	8919,7	1984,4	1982, 503–1986,405	8933,6
Strict.const.est	1988,6	1985, 795–1991,250	8976,0	1983,4	1980, 780–1985,807	8238,7
Strict.const.fix	1989,6	1987, 461–1991,472	8397,2	1983,7	1981, 579–1985,810	8291,1
Strict.expo.est	1989,0	1986, 547–1991,428	9001,0	1983,8	1981, 381–1985,944	7896,2
Strict.expo.fix	1989,9	1988, 027–1991,499	9001,0	1984,2	1982, 241–1986,002	8765,9

Estimates marked in bold correspond to the estimates from the “best fitting” model as was determined through Bayes Factor calculation following 1000 replicates.

Relax – Relax molecular clock; Strict – Strict molecular clock; ESS – Effective Sample Size; HPD – Highest Posterior Density; BSP – Bayesian Skyline Plot tree prior; Const – Constant population size tree prior; Expo – Exponential growth tree prior; est – estimated mutation rate; fix – fixed mutation rate.

**Figure 3:**
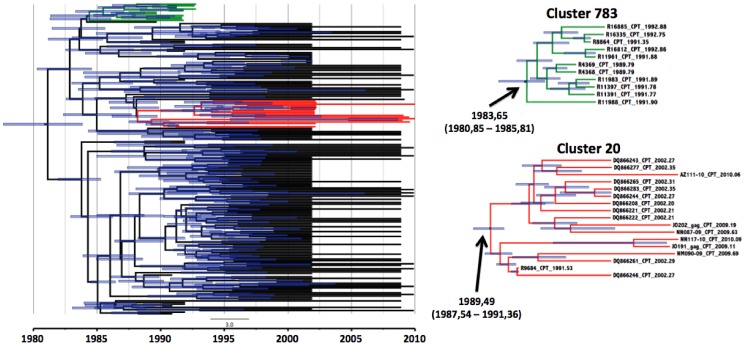
*gag* p24 transmission clusters of Capetonian sequences. On the right hand side are the two individual *gag* clusters with their estimated time of origin. On the left hand side is a big Bayesian time resolved phylogenetic tree with 193 *gag* p24 Capetonian sequences with the two monophyletic clades that were identified in the PhyloType analysis.

**Figure 4:**
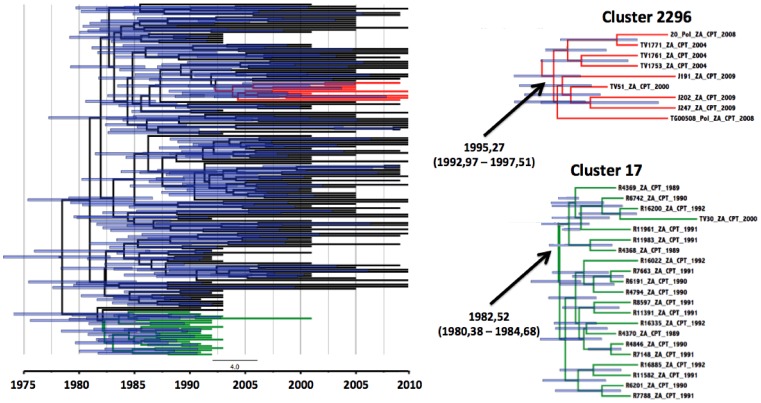
RT-*pol* transmission clusters of Capetonian sequences. On the right hand side are the two individual *pol* clusters with their estimated time of origin. On the left hand side is a big Bayesian time resolved phylogenetic tree with 166 RT-*pol* Capetonian sequences with the two monophyletic clades that were identified in the PhyloType analysis.

The results from the BEAST analysis indicated that these clusters have been circulating amongst isolated parts of the infected population of Cape Town for several years. The estimated dates of origin of the oldest clusters (dating back from 1989–2000), cluster 17 and cluster 783, were identified to be 1983, 43 (95% HPD interval 1980, 78–1985, 66) and 1982,06 (95% HPD interval 1975, 83–1986, 34) respectively. Cluster 2296 and cluster 20 were estimated to have originated around 1996, 27 (95% HPD interval 1993, 48–1998, 74) and 1989, 48 (95% HPD interval 1984, 32–1992, 50) respectively. Time resolved phylogenies of the two complete Cape Town datasets (Figures 3 and 4), constructed through the Bayesian analysis in TreeAnnotator, also confirmed the estimated dates of origin for each of the transmission events as was estimated in standard MCMC Bayesian runs under various models.

## Discussion

Several clusters were observed through manual evaluation of the Bayesian tree topologies for both the *gag* p24 and RT-*pol* datasets. Strict analyses of the inferred phylogenies in PhyloType were identified on the basis of statistical support and high separation/diversity ratio. The results from the two different genomic regions coincided in terms of number of clusters. In total, two clusters in the RT-*pol* dataset (cluster 2296 and cluster 17) and two clusters in the *gag* p24 dataset (cluster 20 and cluster 783) were identified. The posterior support for the clusters in the Bayesian *gag* p24 tree topology was on average lower (0.673 and 0.714) than those for the clusters in the RT-*pol* tree topology (0.891 and 0.815). This, however, can be attributed to the small fragment length of the *gag* p24 dataset in comparison with the RT-*pol* dataset (444 vs 1002 bp). The patient samples that are represented in clusters 783 and 17 correspond well with one another with a total of 4 patients being present in both the two clusters. However, another four of the patients in cluster 783 clustered in an adjacent clade to cluster 17. These two clusters both contain patients from the very early years of the HIV epidemic in Cape Town (1989–1992) and the estimated tMRCA for both clusters was placed around the early 1980's. The other two clusters, cluster 20 and cluster 2296, represent more recently sampled patients from the Cape Metropolitan area with estimated dates of origin around the early 1990's and mid 1990's respectively.

Close examination of all available patient data from each of the sequences, which clustered in the four transmission events revealed that the majority (92,16%) of the patients became infected through heterosexual contact. Two of the patients within the identified transmission clusters became infected through the MSM route while one patient became infected while serving out a prison sentence. We classified the prison transmission as separate from the other MSM infections as the true mode of transmission (heterosexual, MSM or intravenous drug use) is not fully known. The patients in the identified clusters comprise people from diverse demographic backgrounds. The subtype C virus in the Cape Metropolitan region seems to circulate amongst Africans, people from Mixed Race backgrounds as well as amongst a small number of Caucasian individuals.

These identified clusters represent the first identified transmission clusters of HIV-1 in South Africa to date. Until now, only small transmission events of HIV-1 have been reported [37]. Furthermore, these findings supplement previous findings from Gordon and co-workers [38], which observed a strong basal clustering of older samples. Basal clustering of the older samples in our Cape Town dataset was also observed in the present study. There are stark differences between the HIV-1 epidemic in Cape Town and that of the rest of South Africa. Firstly, HIV prevalence trends in the western parts of South Africa, which encompasses the Cape Metropolitan region, have a much lower prevalence of HIV (5.0% compared to 16.9% in the eastern parts of the country) [39]. Secondly, the demographic characteristics of the western parts of the country are also different from those in the eastern parts, with a far more cosmopolitan “make up” including people from Mixed Race, Caucasian, native African and immigrant backgrounds as well as a much higher concentration of the MSM population.

The results from this study are unique in that they show that HIV-1 subtype C was circulating amongst the infected population of the Cape Metropolitan area prior to the first documented cases of heterosexually acquired HIV-1 subtype C in the late 1980's. The HIV-1 epidemic during the course of the 1980's was mainly concentrated amongst the MSM risk group within South Africa, from whom HIV-1 subtype B and subtype D viruses had also been isolated [40]. Furthermore, this study shows the presence of HIV-1 subtype C infections amongst patients who became infected via MSM contact. This illustrates that the epidemic in Cape Town is far more dynamic than was previously thought and that transmission between MSM and heterosexual risk groups does occur. Furthermore, the patients contained within the identified transmission clusters encompass individuals from a diverse demographic and socio-economic background.

## Conclusion

These transmission events represent the first identified transmission events of HIV amongst the general population in South Africa. It is evident that by increasing the coverage of randomly sampled specimens within a defined geographic region, it is possible to start to uncover transmission events of HIV amongst local communities. The identified transmission clusters primarily contain sequence data from patients who became infected through heterosexual contact, which confirms that heterosexual transmission of HIV within South Africa remains the primary mode of transmission of the virus. A small number of patients within the identified transmission clusters became infected through the MSM route or while incarcerated, which indicates that the transmission of subtype C HIV-1 is not exclusively confined to the general heterosexual population of the Cape Metropolitan area. It is, therefore, important that HIV prevention and care not just target the general heterosexual population but targets all people from different demographic, sexual, racial and socio-economic backgrounds. The continued monitoring and testing of the HIV epidemic, including the investigation of the nature of transmission of the virus, is of critical importance in the fight against the HIV/AIDS epidemic, not only within South Africa but also in other sub-Saharan African countries and the world at large.
